# Healing activity of *Casearia sylvestris* Sw. in second-degree scald burns in rodents

**DOI:** 10.1186/s13104-015-1251-4

**Published:** 2015-06-26

**Authors:** Evandro Pedro de Campos, Letícia Nava Trombini, Rafaela Rodrigues, Décio Luis Portella, Adriana Carolina Werner, Miriele Cristina Ferraz, Robson Vicente Machado de Oliveira, José Carlos Cogo, Yoko Oshima-Franco, Norberto Aranha, Marli Gerenutti

**Affiliations:** Laboratory for the Toxicological Research (Lapetox), Department of Pharmaceutical Sciences, University of Sorocaba (UNISO), Cidade Universitária, Rodovia Raposo Tavares km 92.5, 18023-000 Sorocaba, São Paulo Brazil; Serpentarium of the Vale do Paraíba University (CEN-UNIVAP), Av Shishima Hifumi 2911, 12244-000 São José dos Campos, São Paulo Brazil; Department of Technological and Environmental Process, University of Sorocaba (UNISO), Rodovia Raposo Tavares km 92.5, 18023-000 Sorocaba, São Paulo Brazil

**Keywords:** *Casearia sylvestris*, Burns, Biofilm, *Acetobacter xylinum*, *Bothrops jararacussu*

## Abstract

**Background:**

Every year thousands of people are victims of burns, mainly scald burns. Many of these victims have small size wounds and superficial partial thickness and do not seek specialized medical care. As in Brazil *Casearia sylvestris* Sw., popularly known as guaçatonga is widely used for its analgesic, antiseptic and anti-inflammatory activities, this study sought to evaluate the effects of its hydroalcoholic extract in healing process of burns injuries.

**Methods:**

The obtained extract was validated applying a thin layer chromatography and sophisticated validation method using *Bothrops jararacussu* snake venom that is necrotic and inflammatory, and by which guaçatonga extract was able to neutralize the irreversible neuromuscular blockade induced by the venom. After induction of the scald injury, the animals were treated daily with saline solution spray; spray containing extract; biofilm; or biofilm impregnated with extract.

**Results:**

Significant differences were observed between the four groups studied considering: extension of the healing area, neovascularization, fibroblast proliferation, and epithelialization.

**Conclusion:**

The anti-inflammatory and bactericidal effects of *C. sylvestris* Sw. suggests a potential therapeutic benefit in the treatment of inflammatory conditions in second-degree scald burn injuries, as well as, counteracting against the in vitro paralysis induced by *B. jararacussu* venom.

## Background

Perhaps the most traumatic injury to the victim, over the years burns have been treated with various wound coverings, both natural and synthetic aiming at find the most efficient means to control the damage that go beyond the site of the injury.

It is important as well promote healing, since the primary goal when it comes to the treatment of burns is the physiological closure in the shortest possible time [1].

Usually, the wound treatment begins by cleansing, debridement and finally provides a conducive humid ambient to the natural healing process. So, the ideal covering material must: provide a moist environment; serve as a bacterial barrier and be a means to facilitate gas exchange preventing, however, the action of toxic contaminants [2].

Although Badr et al. [3] pointed out that traditional knowledge about different treatments for burns have been extracted from Persian manuscripts, only recently the traditional use of plants for wound healing has received attention by the scientific community [4, 5].

Folk medicine has made use of various plants with anti-inflammatory and healing actions in the treatment of burn injuries and cut wounds [6–8] and *Casearia sylvestris* Sw. (Salicaceae) is one of these plants.

Also known as “guaçatonga”, this term of Tupi-Guarani (Brazilian unwritten indigenous native language) origin indicates an age-old usage of *C. sylvestris* Sw. by Brazilian indigenous communities. Other traditional names are “chá arebugre”, “cafeeiro-do-mato”, “cafezinho-do-mato” and “erva bugre”.

*Casearia sylvestris* Sw. is a shrub that occurs in forests of Southern Brazil. The hydroalcoholic extract of its leaves contains, among the chemical constituents, various diterpenes and triterpenes, hexanoic acid and caproic acid [9, 10].

There are some reports about using *C. sylvestris* Sw. for treating skin lesions and small ulcerations. Studies have shown additional pharmacological properties as analgesic and anti-inflammatory, also to chemotherapeutic potential [11, 12]. De Mattos et al. [13] suggests a possible therapeutic benefit of *C. sylvestris* Sw. in treating conditions associated with inflammatory pain.

Here, the obtained hydroalcoholic extract from *C. sylvestris* Sw. leaves was: (1) validated according to Francischinelli et al. [14] using *Bothrops jararacussu* snake venom, a well-known myotoxic and fiery venom; (2) assayed on scalded rats with second-degree induced burns aiming to evaluate its healing activity.

## Methods

### Preparation hydroalcoholic extract

An amount of 2.95 kg *C. sylvestris* Sw. leaves were dried and grinded, yielding 2.80 kg of the leaves powder. It was used 35.3 L of 70% ethanol to obtain 35 L of hydroalcoholic extract of this powder. Then the ethanol was removed from the extract using a rotatory evaporator, and subsequently lyophilized at −20°C, yielding 522.9 g of the lyophilized powder. It was stored at room temperature without light and moisture until the pharmacological assays were performed.

### Pharmacognostic validation of *Casearia sylvestris* Sw. leaves hydroalcoholic extract (thin layer chromatography—TLC)

Aliquots of hydroalcoholic from *C. sylvestris* Sw. leaf powder was spotted onto 0.3 mm thick silica gel plates (Merck^®^, Germany), along with appropriate standards [15] and compared to methanol extracts [14]. The TLC system for running the extracts consisted of acetone:chloroform:formic acid (10:75:8; v/v), visualized with NP/PEG as follows: 5% (v/v) ethanol NP (diphenyl boric acid 2-aminoethyl ester, Sigma^®^, Switzerland) followed by 5% (v/v) ethanol PEG 4000 (polyethylene glycol 4000, Synth^®^), being visualized under UV light at 360 nm. Comparison with the standards investigated the presence of phytochemical groups, caffeic acid and rutin (all from Sigma-Aldrich^®^, USA) solubilized in methanol (1 mg/mL). The chromatographic profile of *C. sylvestris* Sw. extracts were compared to phytochemical standards.

### Animals

Male Swiss white mice weighing 26–32 g and male adult Wistar rats weighing 200–250 g were obtained from Anilab Laboratory Animal Creation and Trade Ltd., São Paulo State. They were kept in the UNISO/Pharmacy School facilities in accordance with “Guide for the Care and Use of Laboratory Animal” (National Research Council) and the Organization for Economic Co-operation and Development guidance document, approved by the Committee on the Care and Use of Experimental Animals, protocol No. 2011-54P, Brazilian Lutheran University (ULBRA). Each animal was housed individually in micro-environmental compartments free of contamination from outside, with proper individual exhaust fan directly into the cage.

### *Bothrops jararacussu* (Bjssu) venom

*Bothrops jararacussu* venom was collected from two adult specimens kept in the serpentarium of the Centro de Estudos da Natureza—CEN. The venom was lyophilized and certified by Professor José Carlos Cogo, Ph.D., from Vale do Paraiba University, Univap, SP, Brazil.

The experiment was approved by the Ethical Committee for Research of the Vale do Paraiba University, protocol No. A091/CEP/2007. All the procedures were in agreement with the Ethical Principles in Animal Research, adopted by the Brazilian College of Animal Experimentation (COBEA).

### Hydroalcoholic extract of *Casearia sylvestris* leaves validation

The obtained extract was validated using phrenic nerve-diaphragm preparations from rats anesthetized with halothane (Cristália^®^, Itapira, SP, Brazil) and killed by exsanguination. For the extract validation, the mice were divided into four groups of six.

The preparations were mounted as described by Bülbring [16] (modified for mice) and mounted under a tension of 5 g in a 5 mL organ bath containing Tyrode solution with the following composition in mM: NaCl 137, KCl 2.7, CaCl_2_ 1.8, MgCl_2_ 0.49, NaH_2_PO_4_ 0.42, NaHCO_3_ 11.9 and glucose 11.1. After equilibration with the carbogen aeration mixture of 95% O_2_/5% CO_2_, the pH of the solution was set at 7.0. Then they were stimulated indirectly (3 V) with supramaximal stimuli (4× threshold, 0.06 Hz, 0.2 ms) delivered from a stimulator (Model ESF-15D, Ribeirão Preto, SP, Brazil) to the phrenic nerve through bipolar electrodes. Isometric twitch tension was recorded with a force–displacement transducer coupled to a two-channel recorder Gemini physiographic device via a primary preamplifier (all from Ugo Basile^®^, Italy).

The preparations were allowed to stabilize for at least 20 min before incubation with hydroalcoholic extract of *C. sylvestris* (200 µg/mL), concentration that did not cause interference on basal response of neuromuscular preparation [14], Bjssu venom (40 µg/mL), preincubated Bjssu venom (40 µg/mL) + hydroalcoholic extract (200 µg/mL) mixture, for 30 min before addition into the organ bath; or Tyrode solution alone (control). Bjssu snake venom induces an irreversible neuromuscular blockade due to its high myotoxic ability [17].

### Pharmacotechnical development

(A) Incorporation of the extract of *C. sylvestris* Sw. in its liquid pharmaceutical dosage form (q.s. 50 mL): 1.0 g of lyophilized extract of wild *C. sylvestris* Sw. dissolved in 4.0 mL of PEG 400 (Oxiteno): 1.0 mL of Milli-Q^®^ water; 0.01 g of propyl paraben; 0.09 g of methyl paraben; 1.25 g propylene glycol (Ecibra); 0.1 g of EDTA (dissolved in 10.0 mL of Milli-Q^®^ water, at 70°C); pH: 6.0, adjusted with triethanolamine. The dosage of 1.0 g of lyophilized extract of wild *C. sylvestris* Sw. was chosen due to be the most stable concentration in the pharmaceutical formulation.
(B) Incorporation of *C. sylvestris* Sw. extract on biofilm dressings (20% ethanol, highest soluble concentration of lyophilized powder). The biofilm’s dressings (3 cm × 3 cm) produced with *Acetobacter xylinum* from the Institute of Chemistry of Araraquara, São Paulo State University—UNESP, were submerged in solution (20% ethanol) of lyophilized extract of *C. sylvestris* Sw., kept for 48 h at room temperature and dried in stove at 36°C for 6 h. These intervals were defined after tests merging the extract in the biofilm, and of the release of the incorporated extract, by spectrophotometric analysis.

### Induction and treatment of second-degree scald burns

For the induction of injury, the rats were divided into four groups of 10, anesthetized (ketamine hydrochloride: 100.0 mg/kg) and subjected to neuromuscular blockade (6.0 mg/kg xylazine hydrochloride). All animals were epilated in the back (3 cm × 3 cm) and that epilated area placed in contact with water at 70°C (800 mL, in a Becker and kept heated by a heating mantle) for 10 s [18]. The treatment was initiated 30 min after the burn induction.

The four groups were divided and treated as follows: (1) animals in the control group treated daily with 9% saline solution spray (CS); (2) animals treated daily with spray containing extract of *C. sylvestris* Sw (CaS).; (3) animals treated with biofilm (CB); and (4) animals treated with biofilm impregnated with 20% extract of *C. sylvestris* Sw. (CaB). The spray solution applications were standardized at 10 cm distance to the lesion and three jets in each application, and the biofilm treatments had a single exposure.

The daily evolution of the wounds was evaluated macroscopically and was given a score according to its intensity, considering each criterion: 0 (severe infection/great extent of necrosis); 1 (moderate exudation rates/hyperemia); 2 (inflammatory signs/infection edges); 3 (initial epithelialization); 4 (partial epithelialization/absence of necrotic area); 5 (epithelialization/hair growth).

The rats were sacrificed on the 24th day, through of an overdose of the anesthetic ketamine hydrochloride (148 mg/kg) [19] and the burned area was sent for histopathological analysis, without identifying which dressing had been used. Serial sections with an average thickness of 5.0 μm were made.

After deparaffinization and diaphanization, sections were stained with hematoxylin and eosin (HE), Masson’s trichrome (TRI) and orcein (OC). Histological analysis was performed on a microscope (Nikon Eclipse^®^ 200) and photos taken by the NIS ELEMENTS AIR™ 3.0 software. The evaluation criteria were: epithelialization (EPI); extent of healing area based on the lateral extension (EHA); chronic inflammatory infiltration (INF), neovascularization (NEO), fibroblast proliferation (FIB) and young collagen (COL). For each criterion assessed, a score (0–5) was assigned according to its greatest intensity in the histological slide.

### Calculation of the body surface of a Wistar rat

According Gouma [20] the formula for calculating the body surface of a Wistar rat is SC = K × P^2/3^, ranging 3% in total, where SC is the body surface in cm^2^, K is the Meeh constant = 10.65 and P equals the weight of the mouse in grams. Considering the average rat weight 245 g, SC = 10.65 × 245^2/3^, resulting in approximately 416.99 cm^2^ for the total body surface area of rats. Thus, the burn caused in our experiment (9 cm^2^) equals 2.16% of total body area.

### Statistical analysis

The results from pharmacological assays are shown as the mean ± SEM, and they were analyzed statistically using Student’s *t* test. Bartlett test was employed to evaluate the outcomes homogeneity. Differences between each treatment were assessed using one-way ANOVA, followed by Tukey–Kramer’s multiple range tests. Data were analyzed in Instat (GraphPad Software—USA).The significance level was set at 5% for all experiments.

## Results

### Pharmacognostical validation (TLC)

**Figure 1:**
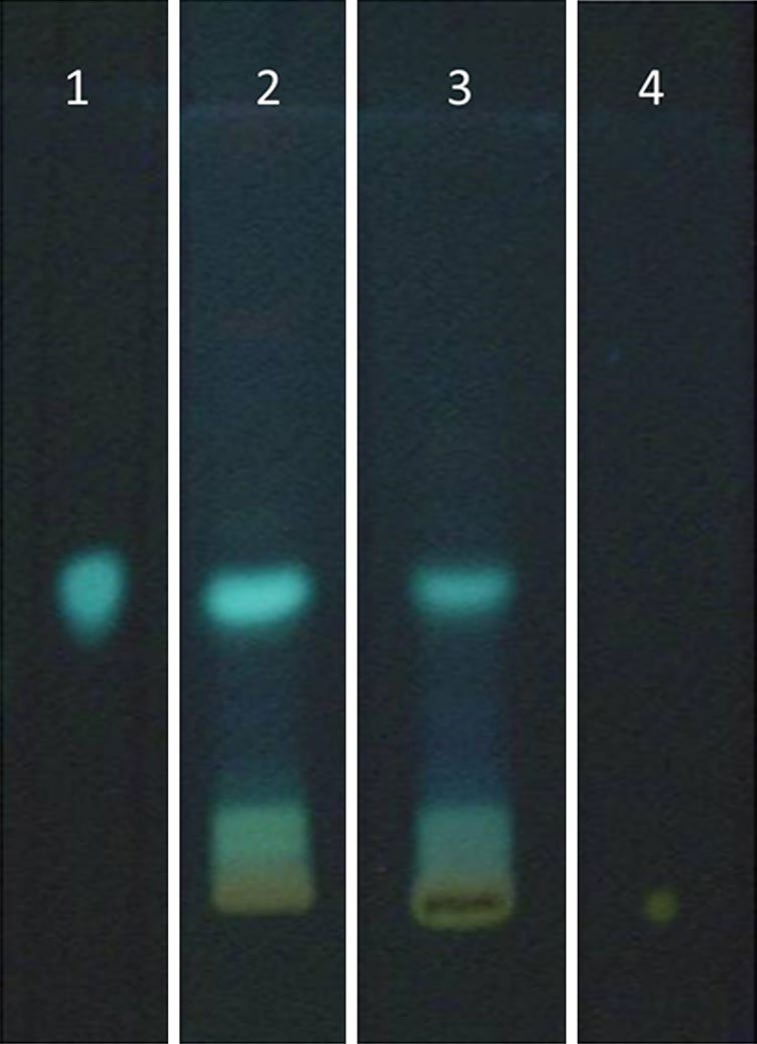
Chromatographic profile of hydroalcoholic (*2*) and methanol (*3*) extracts of *Casearia sylvestris* Sw. leaves by thin layer chromatography. Solvent system: acetone:chloroform:formic acid (10:75:8; v/v). Developer: NP/PEG; NP, diphenylboric acid 2-aminoethyl ester. *PEG* polyethylene glycol. Standards: *1* caffeic acid; *4* rutin.

Figure 1 shows the chromatoplaque of hydroalcoholic extract (2) in comparison to previously obtained methanolic extract (3) from *C. sylvestris* Sw. leaves. Phytochemical standards as caffeic acid (1) and rutin (4) only expressed caffeic acid, in reason of the applied solvent system. Note the similarity between hydroalcoholic and methanol extracts, showing that both extracts represent the same phytochemical substances.

### Hydroalcoholic extract of *Casearia sylvestris* Sw. leaves validation

**Figure 2:**
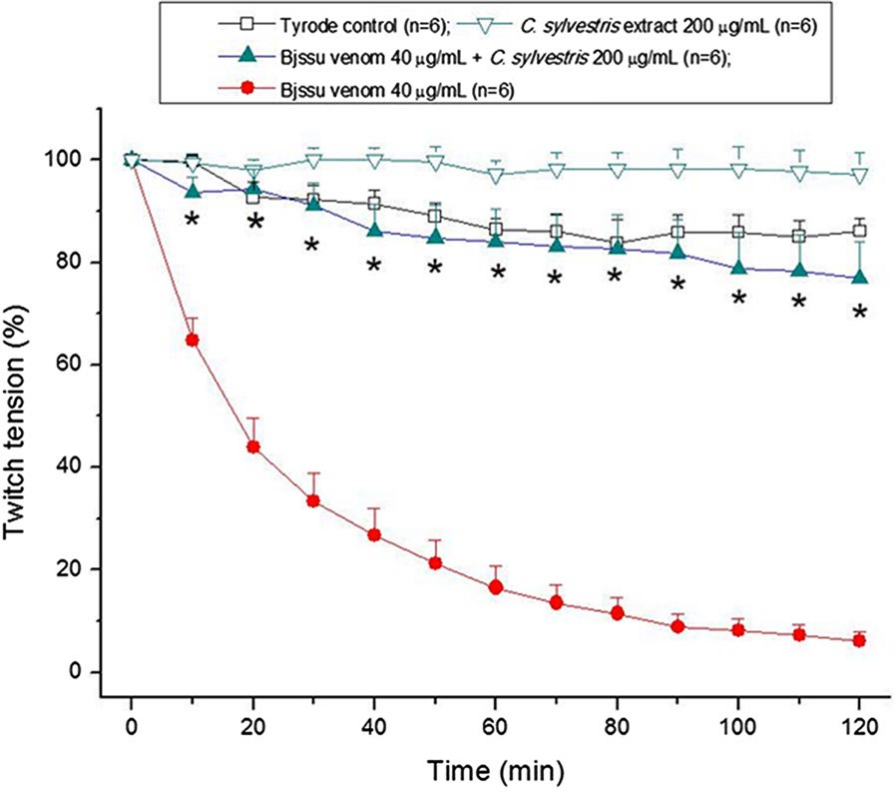
Contractile responses of indirectly stimulated PND preparations incubated with *B. jararacussu* venom (Bjssu 40 µg/mL); hydroalcoholic extract of *C. sylvestris* Sw. (200 μg/mL), and preincubated mixture (30 min) of extract + venom. The *points* are the mean ± SEM of the number of experiments indicated in the *figure* (six animals per group; *p < 0.05 compared to the hatred, Student’s *t* test).

In this set of pharmacological experiments (Figure 2), the Bjssu crude venom was assayed for producing its characteristic irreversible neuromuscular blockade (40 µg/mL). The obtained hydroalcoholic extract from *C. sylvestris* Sw. leaves at 200 µg/mL did not alter the basal response of neuromuscular preparation (p > 0.05 when compared to Tyrode control, but *p < 0.05 when compared to Bjssu venom). Preincubated mixture containing Bjssu venom (40 µg/mL) + hydroalcoholic extract from *C. sylvestris* leaves (200 µg/mL) did entirely protect against the blockade induced by the venom (*p < 0.05 compared to the venom).

### Macroscopic evaluation of the lesions

**Figure 3:**
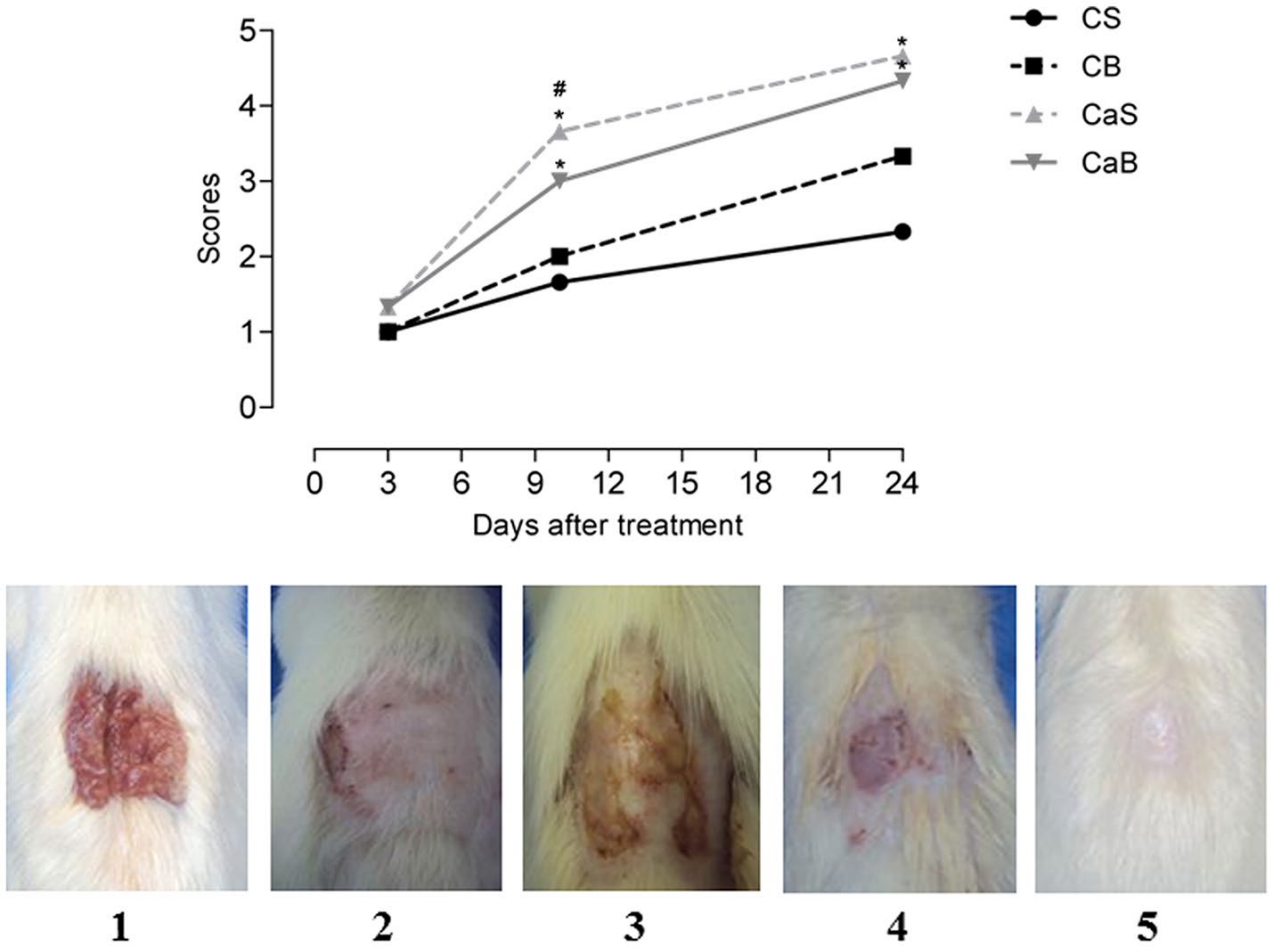
Macroscopical parameters: *C. sylvestris* Sw. effects over the burn injuries. (ten animals per group; *p < 0.05, Tukey–KramerTest). Scores: *1* (moderate exudation rates/hyperemia); *2* (inflammatory signs/infection edges); *3* (initial epithelialization); *4* (partial epithelialization/absence of necrotic area); *5* (epithelialization/hair growth).

Figure 3 illustrates the evolution of macroscopic lesions in the 3rd, 10th and 24th days of treatment, and although there are differences between the groups, no animal developed severe wound infection or other adverse conditions to the healing process. On the 3rd day it was observed no difference between groups (C = F = 0.6667, p = 0.5957), however in the 10th (F = 15.167, p = 0.0012) and 24th day (F = 10.000, p = 0.0044), groups CaS and CaB showed better progression of the healing when compared to CS and CB groups.

#### Histological analysis of the lesions

Histological analysis indicated that all lesions had induced depth of necrosis (variable from the epidermis to the panniculus) compatible with a second-degree burn, and no injury has evolved in the presence of elastic fibers.

**Figure 4:**
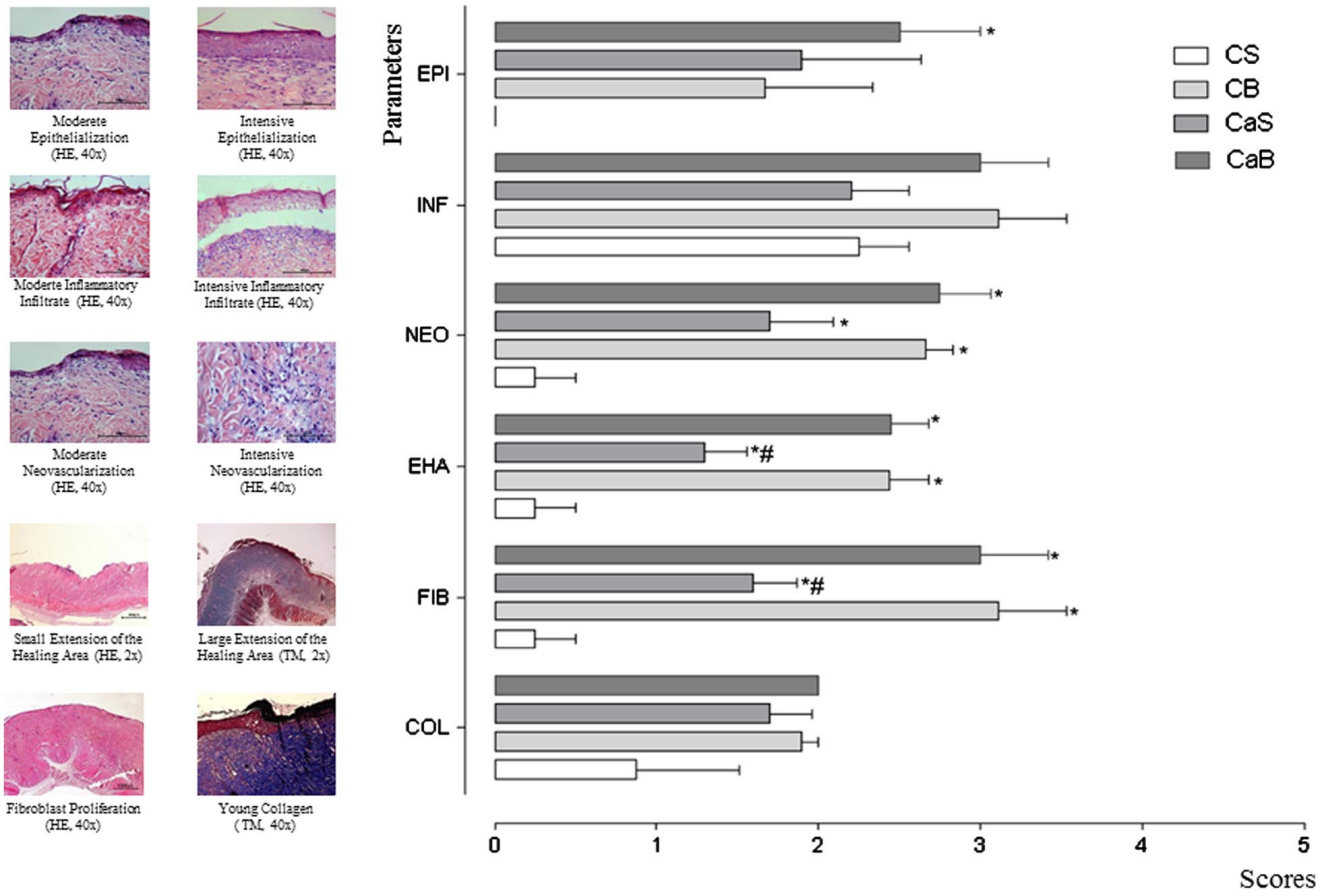
Histological parameters: *C. sylvestris* Sw. effects over the burn injuries. (ten animals per group; *p < 0.05, Tukey–Kramer Test). Scores:* 1* (moderate) to* 5* (intense); *EPI* epithelialization, *EHA* extent of healing area based on the lateral extension, *INF* chronic inflammatory infiltration, *NEO* neovascularization, *FIB* fibroblast proliferation and *COL* young collagen.

Figure 4 shows the evolution of the histological analysis of the lesions, where significant differences were found between the four groups studied for the parameters: extent of healing area (F = 16.706, p = 0.0001); neovascularization (F = 13.662, p = 0.0001); fibroblast proliferation (F = 14.248, p = 0.0001); and epithelialization (F = 3.025, p = 0.0443); no differences were observed between the groups for chronic inflammatory infiltrate (F = 1.589, p = 0.2099) and young collagen (F = 2.296, p = 0.0965).

## Discussion

Burns injuries are considered to be the third leading cause of accidental death in the world, in all age groups, being the most common cause scalding, and the home the environment of greatest frequency of accidents, making up 60% of total occurrences [21]. Alternatives for the treatment of these lesions that contribute to the reduction of pain, time, and epithelialization of the sequelae studies are crucial.

Burn wound healing is a complicated process including inflammation, re-epithelialization, granulation, neovascularization and wound contraction. Several biochemicals are involved in burn healing process including antioxidants, cytokines and liver and kidney damage biomarkers. Phytochemicals represented positive activity at different stages of burn wound healing process by various mechanisms including antimicrobial, anti-inflammatory, antioxidant, collagen synthesis stimulation, cell proliferative and angiogenic effect [8].

In burns, oxidative stress is a perpetuating factor of the inflammatory response, leading to progressive worsening of the metabolic state of the patient. Multiple dysfunction of systems and organs often presents itself as the final complication in severe burns, and probably is related to the sequence of events that take place after the injury. Among these, the excessive production of free radicals plays an important role in the recruitment of inflammatory cells and endothelial dysfunction [22]. Thus, understanding the mechanisms involved in excessive production of free radicals in physiological mechanisms such as phagocytosis, inflammatory reaction and phenomenon of ischemia/reperfusion in individuals who have suffered burns, it is critical for an appropriate therapy.

As shown in the literature, it was not possible uniform scald burns [23], so even though the epilated area has been manually traced and photographed in each 3 days interval, remains hampered the calculation of wound retraction, which is quite accurate in experiments where were used cylindrical metal rod to induce second-degree burn [24–26]. Our studies corroborate Bader et al. [27] where the evolution of the closure of the scald injuries was also evaluated by scores.

On the other hand, development of histological tissues obtained in this study were consistent with observed by Pereira et al. [28], where the reepithelialization time was lower for animals treated with isolectin hydrogel, starting around the burn edge on the 14th day, and the injuries have been completely reepithelialized in 35 days.

Similarly, Akbari et al. [29] evaluated the parameters fibrosis, angiogenesis, infiltration and collagen deposition in animals with second degree burns, wounds were dressed daily with nettle extract, silver sulfadiazine, vaseline and without any medication in control group. Histological scoring was undertaken for scar tissue samples on days 10th and 42nd.

In this sense, was selected *C. sylvestris* Sw. by its well-known medicinal properties [30], including as anti-ophidian [14, 31, 32].

TLC was used for confirming the pharmacognostic profile of hydroalcoholic extract obtained from leaves of *C. sylvestris* in comparison to methanolic extract previously obtained by Cintral-Francischinelli et al. [14]. Both extracts show a similar pattern, which rationale was also showed by the pharmacological assay for confirming the efficacy of hydroalcoholic extract obtained from leaves of *C. sylvestris* Sw., our rationale was to evaluate against the snake venom *B. jararacussu*, which induces myonecrosis leading to an irreversible neuromuscular blockade. As expected, the *C. sylvestris* Sw extract protected (77 ± 7%) against the paralyzis induced by the venom (Figure 2) demonstrating to have the same phytochemical characteristics described in the literature [32] to avoid the myonecrosis evolution. This highlight model adopted can be related to the burn lesion since it involves the same dynamic process in the snakebite envenomation: hemostasis, inflammation, proliferation, and tissue remodeling [33].

Albano et al. [34] observed that the *C. sylvestris* Sw. extract (3–300 mg/kg) acting on the reduction of the inflammatory response to carrageenan was investigated in paw edema in mice. These authors suggest that the anti-inflammatory action of *Casearea sylvestris* SW. due to its ability to inhibit the migration of cells, the enzymatic activity of the myeloperoxidase (MPO) and the production of nitrite/nitrate or edema.

The macroscopic analysis of the lesions suggests that *C. sylvestris* extract Sw. showed a positive result in skin lesions. Similar results were observed in cattle, only during the first 2 days of treatment with the *C. sylvestris* Sw. [35].

It is well established that the inflammatory response following acute UV light irradiation of the skin and the degenerative processes related to chronic UV irradiation skin exposure are largely mediated by the overproduction of reactive oxygen species (ROS) and free radicals and by the impairment of antioxidant systems [36].

The differences between the development of the healings observed in animals treated with saline and *C. sylvestris* Sw. solution, may be attributed to the presence of rutin, which have powerful antioxidant properties, and is one of the components in the extract of *C. sylvestris* [21]. The rutin is a flavonoid with potent antioxidant action and already reported as possessing anti-inflammatory and anti-allergic properties, as well as the capacity of inhibiting phospholipases A2 [37] and the hyaluronidase activities [38, 39].

The antioxidant properties of rutin seems promising to attenuate the effects of uncontrolled production of free radicals in burns, leading to the possible decrease in oxidative stress and consequently reducing the risk of infectious complications, improved healing, less worsening of the tissue damage, reduced lipid peroxidation. Moreover, the rutin principles are used in skin burns for the improvement of capillary permeability [40, 41]

Furthermore, in our studies we found that the biofilm produced with *Acetobacter xylinum* by itself is already a dressing that positively contributes to recovery from injury, however when combined with hydroalcoholic *C. sylvestris* observe a significant growth in the epithelialization.

## Conclusions

Overall the results suggest that the hydroalcoholic extract of leaves of *C. sylvestris* Sw. displays healing effects in second degree’s scald burns, which are probably brought by inhibition in the production of inflammatory mediators. The anti-inflammatory and antiseptic effects of hydroalcoholic extract from *C. sylvestris* Sw. suggests a potential therapeutic benefit of this plant in the treatment of inflammatory conditions, also described in *B. jararacussu* envenomation.

## Abbreviations

Bjssu: *Bothrops jararacussu* venom
CaB: biofilm impregnated with extract
CaS: spray containing extract
CB: biofilm
CS: saline solution spray
COL: young collagen
EHA: lateral extension
EPI: epithelialization
FIB: fibroblast proliferation
HE: hematoxylin and eosin
INF: chronic inflammatory infiltration
TLC: thin layer chromatography
NEO: neovascularization
NP: diphenylboric acid 2-aminoethyl ester
OC: orcein
PEG: polyethylene glycol
TRI: Masson’s trichrome
